# “There hasn’t been a career structure to step into”: a qualitative study on perceptions of allied health clinician researcher careers

**DOI:** 10.1186/s12961-021-00801-2

**Published:** 2022-01-09

**Authors:** Caitlin Brandenburg, Elizabeth C. Ward

**Affiliations:** 1 Centre for Functioning and Health Research, Metro South Health, Queensland Health, Brisbane, Australia; 2 School of Health and Rehabilitation Sciences, The University of Queensland, Brisbane, Australia

**Keywords:** Research capacity, Allied health, Health services, Career, Clinician, Research personnel

## Abstract

**Background:**

There are many demonstrated benefits for health service organizations engaging in research. As a result, growing numbers of clinicians are being encouraged to pursue research as part of their clinical roles, including in allied health (AH). However, while the benefits of having clinician researchers embedded in AH services have been well established, the career needs of those engaged in these dual roles are poorly understood. The aim of this study was to examine perspectives of the career pathway for AH clinicians engaged in “clinician researcher” roles within Australian health services.

**Methods:**

A qualitative descriptive study was conducted, utilizing semi-structured interviews. Purposive sampling was used to ensure selection of varied locations, professions and role types. Results were analysed using thematic analysis. Trustworthiness was established using regular peer debriefing during theme development, and respondent validation of final themes.

**Results:**

Fifty-seven AH clinician researchers, including those who did and did not have research as a formal component of their current role, participated in semi-structured interviews. Key themes were as follows: (1) clinician researchers prefer roles which are embedded in health services; (2) current opportunities for clinician researcher roles in health are insufficient; (3) there are deficiencies in the pathway for clinician researcher careers; (4) clinician researchers are not always valued or incentivized by health services; (5) the current career challenges impair the viability of clinician researcher careers; and (6) the clinician researcher career path has been improving, and there is hope it will continue to improve.

**Conclusion:**

This study outlines a number of weaknesses in the current career structure and opportunities for AH clinician researchers in Australian health services. In particular, while there are strong intrinsic drivers to pursue this dual career, extrinsic drivers are poorly developed, including a lack of job opportunities, an unstable career pathway and a lack of valuing or incentivizing this career choice within health services. This often means that clinician researchers feel compelled to choose between a research or clinical career, leading to loss of this valuable combined skill set. The findings of this research may assist health services in developing and supporting improved clinician researcher career pathways.

**Supplementary Information:**

The online version contains supplementary material available at 10.1186/s12961-021-00801-2.

## Background

Studies have shown that health services that are more research-active tend to have lower mortality rates, greater organizational efficiency, better staff retention, and higher patient and staff satisfaction [1–3]. Additionally, the literature is clear that research driven by, or including, clinically active investigators results in improved translation of results and better patient outcomes [1–5], as clinicians are able to utilize their experience to shape patient- and health service-relevant research [4]. The valuable role of clinician researchers in United Kingdom and Australian research translation centres has been emphasized, as well as the need to further support and develop this workforce [6]. At a national level in Australia, “clinician researcher capability” has been identified as one of the priorities of the Medical Research Future Fund [7], and was highlighted as a key target for research capacity-building in the 2012 McKeon Review [8]. However, with significant pressure on public health systems in the context of a growing and ageing population, finding the resources to support research engagement of clinicians is a recognized challenge [9].

The allied health (AH) professions comprise the third largest workforce within healthcare [10], and across these professions, research- and evidence-based practice is a strong focus. An analysis of competencies expected of new graduates of speech pathology, physiotherapy and dietetics degrees in Australia found that around one third of competencies were related to research and evaluation, although these focused primarily on using, rather than producing, research [11]. AH clinicians have also been shown to have positive perceptions of engaging in research. An Australian study including 301 AH professionals working in Victoria found that most (69%) identified themselves as research-active. Of those who were not research-active, the vast majority indicated they would like to participate in research in the future [12].

However, despite strong clinician interest and demonstrated benefits, many studies have identified a number of significant barriers to AH clinician engagement in research [7]. A systematic review of factors that affect AH research culture and capacity in 2016 revealed that lack of time to do research is the most frequently cited barrier, but lack of skills and support are also common [7]. Recognizing that there are such challenges, there is a growing body of evidence on strategies to engage full-time clinicians in research. Australian studies have reported considerable success in improving AH research culture, especially in the state of Queensland [13, 14]. However, whilst such strategies have been successful in supporting clinicians to engage in small amounts of research as a novice, there is little known about what is needed to develop and support individuals wishing to pursue a combined clinical and research career.

There is little consensus on what constitutes a “career” as a clinician researcher, and various terms have been used to label these professionals, including clinician researcher, clinical academic, clinician scientist and practice-based researcher [15]. Paquin [16] found current definitions wanting, and asked, “How much therapy research does a therapist have to do in order to be a clinician-researcher? How much clinical work does a therapy researcher have to do in order to be a clinician-researcher? And how integrated do these activities and roles need to be in the life of a clinician-researcher in order for one to identify as such?” (p. 228). Newington and colleagues tackled this issue in 2021 by interviewing clinical academics in the United Kingdom to analyse their opinions on “what is a clinical academic?” [15]. Most of their participants felt that the term should be used to describe those engaged in providing clinical care and conducting their own research (in contrast to conducting others’ research). Furthermore, some participants felt that the definition of “clinical care” was not necessarily synonymous with a patient-facing role, but could include management and indirect impacts on health service delivery [15].

In Australia, there is a small but growing group of health services that have employed staff in clinical research positions as a way of increasing research engagement among their staff [14, 17]. Having staff holding dedicated clinical research positions within health services has been shown to have multiple positive impacts. A systematic review which focused specifically on health service-embedded research positions revealed clear benefits, including increased funding, increased research activity/outputs, improved research skills and improved research culture [17]. A recent qualitative study similarly highlighted the positive benefits of dedicated clinical research positions, including clinician skill development, increased research activity, clinical and service changes, increased research outputs and collaborations, enhanced research and workplace culture, improved profile of AH, development of research infrastructure, and professional development of individuals in the research positions [18]. Furthermore, in a cross-sectional study in Victoria, Australia, the presence of a “research lead” position was found to be associated with more research activity and better self-reported research success at the organizational and team level [19].

Although the benefits of dedicated research positions within health services are acknowledged, this is still an emerging workforce within health. In the United Kingdom, the clinician academic workforce is reportedly only 0.1% of the total nursing, midwifery and AH professions workforce [20]. The exact size of this workforce in Australia is unknown; however, a study of Victorian AH clinicians revealed that only 36% felt they had access to someone in a self-defined “research lead” position [19]. As a small and relatively new type of position within the Australian AH context, it is then not unexpected that the career pathway for individuals engaged in these roles is unclear. Previous research has touched upon the need for professional development opportunities, job stability and job satisfaction for staff in clinician researcher positions, but this has not yet been fully explored [17]. Case studies of single positions have also outlined some of the challenges for incumbents, including time demands, lack of awareness of role, feeling isolated and a sense of being in the middle of two contrasting worlds [21, 22]. A qualitative study conducted in the United Kingdom which explored challenges in clinical academic careers for nursing, midwifery and AH professionals revealed multiple challenges, including low pay for PhD stipends, instability, lack of time for research, a lack of value of research by health services, paucity of hybrid roles, anxiety about future career directions and a lack of clear career paths [23].

A lack of clear career structure and support is a critical factor that can place clinician researcher careers at risk of failure. Although at present there is limited research into this topic, it is recognized anecdotally that many of those engaged in dual positions have difficulty balancing both clinical and research aspects, and this may ultimately lead them to leave such positions. This issue, and its associated negative consequences for the health workforce, was recently highlighted by the Australian Medical Research Future Fund, which acknowledged that “Many health care professionals have had to choose between life as a researcher or a clinician. This can mean that research does not address problems seen in clinical practice” [8]. The contribution of career structure to this problem has also been highlighted in the United Kingdom: “early career clinical academics face uncertain career paths, and may choose the comparably stable worlds of clinical practice where their skills are in high demand, or a dedicated academic career” [24, p. 9]. In other research, it has been noted that the considerable barriers to pursuing a clinician researcher career meant that those that did so needed considerable self-motivation, but they ultimately felt pressured to choose one path or the other [23].

There is an identified need for a well-developed career pathway to support clinician researchers in AH. A recent rapid review which explored frameworks for embedding research culture in AH practice found that a key enabling factor at the organizational level was the establishment of research career pathways [25]. A separate systematic review of research capacity-building frameworks for AH practitioners also noted that the need for research career pathways was one of the 17 common themes across the six frameworks reviewed [26]. However, aside from identifying that career opportunities and career pathways are important to the future success of this workforce, there has been little exploration of how this occurs in practice. Considering the value clinician researcher positions bring to health services, systematic research is required to help understand what is needed to support the career needs of people who wish to engage in these roles. Once these have been more fully understood, strategies can then be developed that will help support, build and sustain the careers of those wishing to be part of the clinician researcher workforce within health.

The aim of this study was to examine perspectives of the career pathway for AH clinicians engaged in “clinician researcher” roles within Australian health services.

## Methods

### Research design

A qualitative descriptive approach was employed, as defined in Bradshaw [27], utilizing semi-structured individual interviews to collect data. A qualitative descriptive approach is best suited for studies where the aim is to describe a phenomenon or process from the perspectives of the people involved [28]. It generally focuses on a low level of data interpretation, valuing subjective viewpoints as valid truths which do not require further interpretation [27, 29]. For this reason, it was chosen as the most suitable approach to capture the subjective experiences and opinions of AH clinicians engaged in research roles. This research was conducted and reported in accordance with the consolidated criteria for reporting qualitative research (COREQ) checklist [30].

### Participant sampling and recruitment

Participants were recruited through an expression-of-interest email distributed though research and clinical networks in Australia during mid- to late 2020. These included a network of health service-based AH professors and an AH research fellows network. Prospective participants were required to contact researchers to receive a participant information and consent Form. All participants gave individual consent to participate. Purposive sampling was used to recruit participants from a range of Australian states.

For this study, a clinician researcher was defined as someone who has an AH degree, works at least part time in a healthcare delivery setting, and conducts research as an investigator (as opposed to a research assistant). It was not necessary for them to be engaged in patient-facing work, or for their two role types to be integrated into a single position. For these reasons, the inclusion criteria were twofold:

1. Employed in a health service in Australia as an Allied Health Professional (as defined by Allied Health Professions Australia [10]), and
2. Research-active, defined in this context as clinicians that are actively engaged in leading clinical research—either as part of a defined component of current employed position (e.g. conjoint research fellow), or through unfunded or funded short term opportunities (e.g. unfunded projects, projects funded by short term grants).

Both data saturation and purposive sampling criteria were used to determine sample size. Data saturation was defined as per Grady [31]: "New data tend to be redundant of data already collected. In interviews, when the researcher begins to hear the same comments again and again, data saturation is being reached" (p. 26). Data saturation was determined by the primary interviewer and discussed with the research team to confirm. After an initial data collection period, it was determined by researchers that data saturation had been reached for participants from the state of Queensland; therefore, recruitment from that point employed purposive sampling to target participants from other states.

### Data collection

Participants completed individual interviews, conducted by a study investigator (CB) with a background in clinical research and experience in qualitative interviewing. Both investigators were Queensland-based speech pathologists with experience working in health service clinician researcher positions. Interviews were semi-structured, based on the interview schedule provided in Additional file 1. This interview schedule was developed by the investigators, then piloted and refined with a clinician researcher before use. Interview questions were sent to participants for reflection prior to the sessions. Basic participant characteristics were collected at the beginning of the interview, including profession, length of time in profession, length of time in role, type of role (conjoint, etc.) and type of health service (metropolitan, regional, etc.). Interviews typically took 25–35 minutes and took place via secure videoconferencing software.

### Data analysis

Interviews were audio-recorded and transcribed verbatim. Transcripts then underwent thematic analysis using an inductive approach, following the approach outlined in Braun and Clarke [32]. Data analysis was completed by a single investigator (CB) using NVivo software, with regular team meetings to discuss and refine emerging themes. A draft list of generated themes was returned to the participants by email for respondent validation. Final definitions and naming of themes were agreed upon by all team members. Exemplar quotes were chosen for each theme, and any identifying information (e.g. participant's health service or exact job title) was redacted to maintain participant confidentiality.

## Results

### Participant demographics

A total of 57 clinician researchers participated in the study, and their demographic data are shown in Table 1. Respondents were from four states in Australia, with over 60% from Queensland. There were eight AH professions represented within the sample, the largest proportions of which were speech pathologists at 40.4% and physiotherapists at 28.1%. Just over half (52.8%) of the participants had completed a PhD or research master’s degree, and a further 36.8% were currently enrolled in one. Participants were mainly from public hospitals (91.2%) which either served adults only or had mixed caseloads (adult and paediatric populations). In terms of geographical location, 91.2% worked in metropolitan areas. Three quarters (75.4%) had worked in their professional field for > 10 years, and 85.9% of participants had been employed in their current main role for less than 10 years.

**Table 1:** Participant demographics

Participant characteristics	No. (%)
*State*	
Queensland	37 (64.9)
New South Wales	10 (17.5)
Western Australia	6 (10.5)
Victoria	4 (7.0)
*Profession*	
Speech pathologist	23 (40.4)
Physiotherapist	16 (28.1)
Social worker	5 (8.8)
Dietician	4 (7.0)
Occupational therapist	4 (7.0)
Pharmacist	2 (3.5)
Radiation therapist	2 (3.5)
Other	1 (1.8)
*Research higher degree status*	
None	6 (10.5)
Enrolled in research master’s programme	2 (3.5)
Enrolled in a PhD programme	19 (33.3)
Completed research master’s and enrolled in PhD programme	1 (1.8)
Completed research master’s degree programme	1 (1.8)
Completed PhD programme	27 (47.4)
Completed research master’s and PhD programmes	1 (1.8)
*Type of health service*	
Public	52 (91.2)
Private	5 (8.8)
Paediatric	8 (14.0)
Adult	33 (57.9)
Mixed paediatric and adult	16 (28.1)
Metropolitan	52 (91.2)
Regional	5 (8.8)
*Years in profession*	
1–5	3 (5.3)
6–10	11 (19.3)
11–15	11 (19.3)
16–20	11 (19.3)
21–25	13 (22.8)
More than 25	8 (14.0)
*Years in current role*	
1–5	28 (49.1)
6–10	21 (36.8)
11–15	5 (8.8)
16–20	3 (5.3)

A subgroup of 18 participants replied to the respondent validation email. Of these, 14 confirmed that the themes as written reflected their experiences, and four offered minor clarifications or further comments. No themes were changed as a result of the respondent validation; however, three of the participants’ comments resulted in additions to the descriptions of the themes, providing further information or counterpoints. These are specifically identified in the text.

### Thematic analysis

**Fig. 1:**
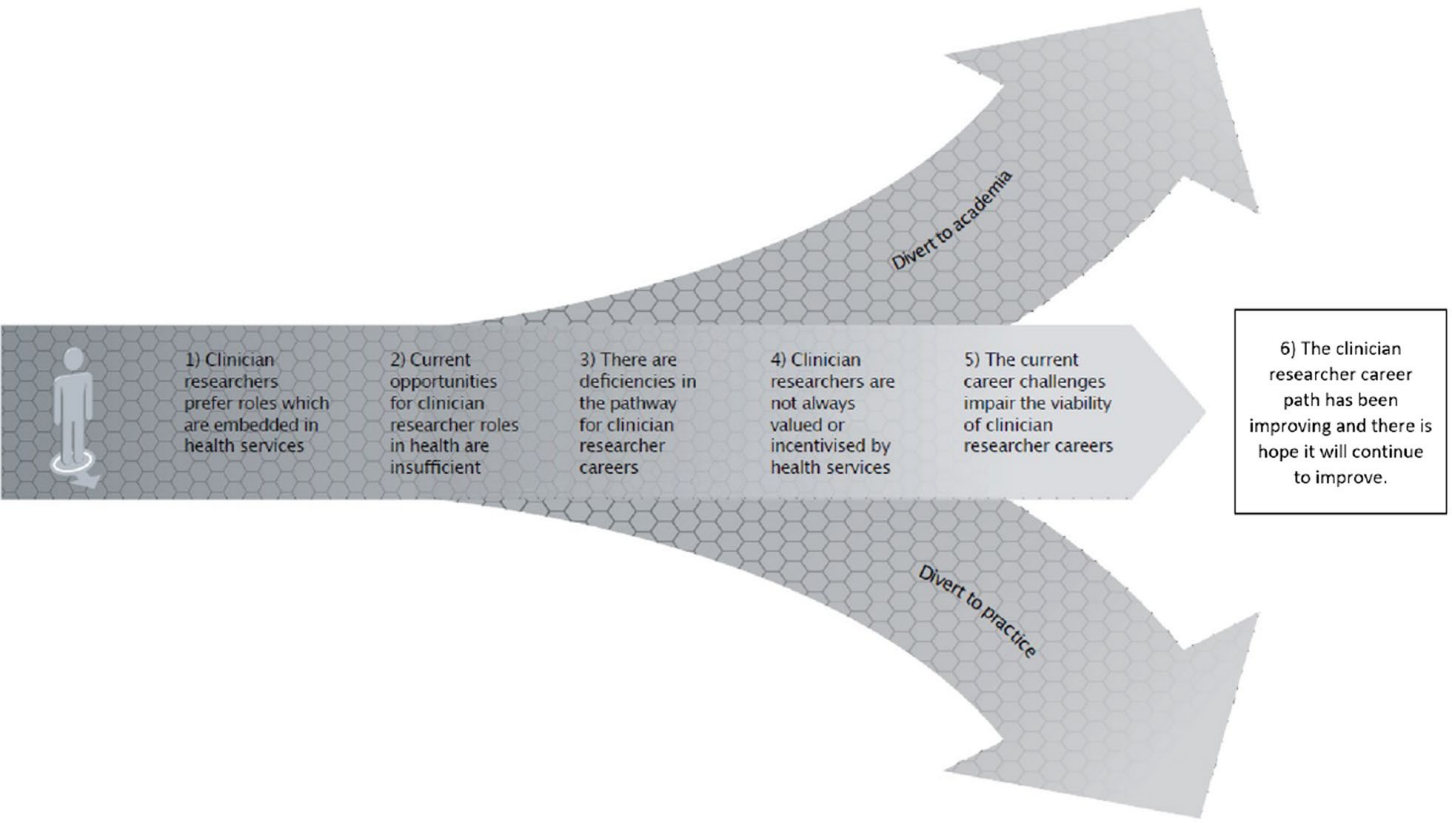
Thematic diagram outlining the six key qualitative themes

The thematic analysis resulted in six nonhierarchical main themes with a total of 14 subthemes. The six main themes are represented diagrammatically in Fig. 1, and consist of the following: (1) clinician researchers prefer roles which are embedded in health services; (2) current opportunities for clinician researcher roles in health are insufficient; (3) there are deficiencies in the pathway for clinician researcher careers; (4) clinician researchers are not always valued or incentivized by health services; (5) consequences of the current career challenges; and (6) the clinician researcher career path has been improving, and there is hope it will continue to improve. Each of these six themes and their subthemes are described in more detail below, and are supported by the exemplar quotes within Table 2.

**Table 2:** Exemplar participant quotes for each subtheme

Theme	Subtheme	Exemplar quotes
Theme 1: Clinician researchers prefer roles which are embedded in health services	Clinically active researchers would prefer to maintain links to clinical practice	“I'm a clinician first. And research for me needs to be clinical and at the forefront. … I guess it's the patient that drives me, not the research.”—P33 “The reason that I want to stay in health is because I love seeing patients, that's what I find really motivating.”—P10
	Clinician researchers are most effective when embedded in healthcare settings	“I feel like health has the huge advantage of you're where the action's happening, you know what the issues are and why they're an issue, because either you're a clinician on the ground or you go to the meetings where they talk about the pressures of the health service.”—P1 “I really believe that the health service-based researchers are in a much better position to have an impact on the people for which the research is designed to help. They are in a much better position to create change in the health system, to translate research into practice, to have a beneficial impact on the community.”—P20
	There are a variety of preferences for combining clinical and research components within a position	“I think having some accepted clinical time to do some research within a clinical space, I think would be sort of what I class a dream position.”—P44 “I'd happily just do a 1-day-a-week clinical role. And then a day a week considering some of that research capacity-building, and then 3 days is actually doing your hands-on research.”—P3
Theme 2: Current opportunities for clinical researcher roles in health are insufficient	Combined clinical practice and research positions are not readily available	“There's pockets of research positions [in health services]. But they're so few and far between.”—P41 “Not from my discipline I don't see that… it's still quite a segregation. It's not an integration within the one role.”—P22
	Clinician researchers often have to make their own job opportunities	“It's all accidental or people creating their own opportunities to be honest…It takes a lot of drive, passion and commitment from the clinician to create the opportunity.”—P30 “I think it's often people just muddle it together themselves, and then departments have been really flexible and accommodating the individual people, as opposed to being seen as a standard way of being professional and within the department potentially.”—P45
Theme 3: There are deficiencies in the pathway for clinician-researcher careers	Research expectations of different levels are unclear	“People have said, just make sure it's not taking up all of your time. But how much time is too much time? Like, is an hour a week too much? Is that enough? I don't know.”—P2 “It's very specific to me as a person that there's an expectation that I continue on [with research], …but there’s not an equitable approach to that across the senior staff … across the service, there's different expectations of that same senior level.”—P13
	There is an absence of dedicated clinician researcher career structure in health	“There hasn't been a career structure to step into. You're either a researcher or you're a clinician or you're a manager. It doesn't feel at this point [that AH disciplines] support any other kind of role description or role type.”—P15 “I can't see a pathway or structure unless you create that pathway … I don't know that there is a pathway that goes, ‘We recognize you as a clinician and we also recognize you as a researcher, and we'll promote that.’”—P21
	Opportunities for progression are limited in current awards	“If you are looking at career progression, I've probably progressed to the point where clinically and even from a research perspective, that's as far as it's going to go… So I guess you'd just be travelling horizontally with a greater research component.”—P39 “At the university you can progress with your career. You can apply for an associate professorship and a professorship. It's not dependent on, ‘Oh, sorry, we've already got eight professors; we can't have another one.’ It's not dependent on roles; it's dependent on you and your achievement.”—P5
	The career pathway has gaps at some levels, particularly post-PhD	“You do—definitely, I do feel quite different post-PhD, because when I was doing my PhD, it was just so much focus on this is the next goal, this is the next milestone. And then when you get your conferral, you'll just resume your normal clinical job. There is a real, ‘Well, what next? What next?’”—P13 “When I started my PhD, I was going in it with rose-coloured glasses and thinking, ‘Oh, there can be all these opportunities for me with my career once I've got a PhD,’ and I'm actually finding that's definitely not the case.”—P28
Theme 4: Clinician researchers are not always valued or incentivized by health services	Recognition and value of research experience in recruitment is variable	“I think it depends on who's doing the recruiting… my feeling is that it's patchy. That in some places someone's research capability does get considered as part of a role description, but in other parts it’s more lip service paid to that.”—P36 “I think there's not a lot of incentive for people to do things like research higher degrees or research in terms of, for example, [senior/advanced level] job descriptions. Having a research higher degree is advertised as being desired, but it's not essential, or I don't know that it's given any additional weight.”—P10
	Clinical research opportunities are generally less well paid and less stable than clinical roles	“If I went into a research position, I'd have to take a downward slide. And so, for me, it's better from a career point of view as well as a financial point of view to stay where I am and do research on the side.”—P24 “There are few research positions I'm aware of in Queensland Health that are substantive, full time, not defined by a 3-year grant or a 3-year contract.”—P5
	The role of clinician researchers is not always valued and enabled in health services	“I’m not entirely convinced [research is valued]. But the health service is always happy to take the glory of the number of papers published and the number of posters …So they're happy to take the glory, but not always happy to support it.”—P34 “There are the exhausting, ongoing issues within the system of health that really are barriers to research … one example is the issues around restricting travel for researchers…That results in that perception of researchers not being valued. So the work that they're doing is not valued.”—P20
Theme 5: The current career challenges impair the viability of clinician researcher careers	Clinician researchers either leave health services to work in academia or become clinicians with little opportunity to do research	“They end up at universities, and I know a couple, maybe one person, has gone back to clinical work because they didn't like research. So I guess that kind of defeats the purpose of having someone have a PhD in hindsight.”—P40 “If I'm going to want to continue my career in research, I either have to go back to being a clinician and do it in my own time or leave and go to a university.”—P28
	Clinician researchers are unable to use their full skill sets, negatively impacting both research and patient care	“I spent 20 years doing clinical before I then started on my research career. And unless I'm still in clinical I feel as though 20 years’ worth of clinical expertise sort of gets wasted to some extent … if you're faced with having to choose between a research career or clinical career, then one or the other is gonna lose out; you're not using the full array of your skill set.”—P15 “There is a lack of where do you go from here. Do I want to totally just lose the clinical skills that I've got and all the expertise in my allied health discipline and become a researcher, or do I want to just have the title ‘doctor’ and be a highly specialist clinician who uses evidence-based practice but doesn't do any research. It's a bit of a tension.”-P15
Theme 6: The clinician researcher career path has been improving, and there is hope it will continue to improve		“Five years ago, I don't feel that if I wanted to do research, I would be supported. I do feel like when I finish my PhD, I would be supported to do clinical research.”—P12 “I think things are progressing fairly quickly. So, I'm optimistic that I might get my dream job one day.”—P9 “I have started to see the trajectory go up over the last 5 years, and I'm aware of different strategies and plans that are in progress…it takes time for things to change and opportunities to develop. Its not like there's going be a huge amount of positions in 5 years, but I think there will be more opportunities over time.”—P7 “I think that culture is changing, these positions are starting to emerge. And they're starting to be developed to acknowledge the connection between research and clinical practice and to acknowledge those types of options as being really important. But I don't think it's easy. I don't think it's clear.”—P48

#### Theme 1: Clinician researchers prefer roles which are embedded in health services

The participants in this study, all of whom worked at least partially in a healthcare delivery setting, expressed a preference to continue to work in this type of setting. As P6 stated, “I don't think I would look outside of health. That's where I am and that's where I'm going to keep going.”

When discussing their preference to stay within health services, participants who were clinically active spoke about the personal fulfilment of working with patients and engaging their clinical skills. Many identified strongly with being a clinician and did not want to lose that identity or feel they were wasting their skills by leaving their clinical role. They spoke about the joy they found in their clinical work, and that their interest in research was often secondary to, or driven by, their patient work. As a consequence, many stated that they would not consider working in another setting such as a university. For others, they stated that they would consider working in a university setting, but this was either not preferred or would need to be combined with a part-time role in healthcare. It was also noted by a few participants that roles in health other than direct patient contact, like clinical education or applied research, could be sufficient to help maintain their feeling of connection to clinical care. As P21 described, “Even if it's not specifically clinical practice, it would be very close proximity to clinical practice, so you really understood what happens on the ground.”

A complementary reason for the preference to work within health services was the value that clinician researchers felt being embedded in a healthcare delivery setting brought to their research, patients and the community. P21 asserted that “having a proximity to patients and clinical care, and the value that has in research and that translation of knowledge is really important.” Participants felt that by being embedded in the healthcare setting, they were able to identify research questions which were most important to practice. Most importantly, they emphasized the value that having a clinician researcher role brought to translating research into practice.

Participants also stated that their knowledge of the health service meant they were able to design research which was more practical to conduct in this setting, which some felt was lacking in academia-generated research. Some participants, like P40, also felt that their role as a researcher enhanced their role as a clinician: “I think doing research makes me a better clinician as well.”

While participants expressed a clear preference for continuing to work in clinical research roles in healthcare, they had a wide variety of preferences of what their ideal position would look like. When asked specifically to identify a “dream role”, a common pattern of response was for a balance between research and practice, ranging from primarily clinical roles with a small amount of dedicated research time embedded, through to majority research roles with an opportunity to keep up clinical skills. Some participants felt that an ideal position would include other roles like research capacity-building, clinical education, teaching and management. There were mixed opinions on whether each component should be flexible or should have a dedicated FTE associated with it (e.g. 0.4 research, 0.6 clinical). On the whole, participants emphasized the importance of having some level of university linkage within positions, but did not have a strong preference for the level of this linkage (e.g. conjoint, adjunct, informal links).

#### Theme 2: Current opportunities for clinician researcher roles in health are insufficient

In Theme 2, participants spoke about how current opportunities for clinician researcher roles in health are insufficient, especially in light of the demand described in Theme 1. As P1 stated, “There's just not many opportunities in health to have a position where research is your job.”

Participants felt that integrated clinical practice and research positions were very rare, and some stated that their preferred role did not currently exist. Many also felt that the availability of positions was better in some states (i.e. Queensland), in urban regions, or within specific departments of their own health service. For example, P53 said, "I don’t think such a job exists in Western Australia", and P17 noted during respondent validation that finding such positions was “impossible” in rural and remote areas. Some clinician researchers also felt that the positions that were available were not always desirable to them, as they were often focused on research capacity-building or joining an established project. P23 said, “I don't know of any roles like that…where you can do your own research ideas…I would only want to do research if it's something that I'm interested in and I can see a benefit from.”

Clinician researchers also felt that because combined roles were not readily available, it was left to individuals to create their own opportunities. Most clinician researchers “cobbled together” (P11) their roles informally through multiple part-time positions or acquisition of highly competitive grant funding. Participants noted that individuals had to be highly driven and self-motivated to achieve this. As P19 noted, “If a clinician does want to do research, it has to be working extra hours in their own time to submit grant applications. There's certainly management support for once a grant application is approved…but in that preparation phase there's no support.” P5 noted that the constant need to both negotiate release from clinical duties and seek out funding to support their own research position “take(s) away from delivering on actual research”.

Even where true combined clinician research positions existed, participants felt that they were often created ad hoc simply because of the passion of one individual, enabled by interest from their manager. This had negative implications for sustainability and succession planning, as P33 noted: “Because people themselves have made them … [the positions] are there because of the person who's in them, more than they're there for someone to work their way into.” Furthermore, when clinician researchers are unable to secure their own opportunities, they often ended up pursuing research in their own, unpaid time. As P4 said (with a sarcastic tone), “[Opportunities exist] if you don't like sleep, and you are happy working nights and weekends, which is how people do it at the moment.”

#### Theme 3: There are deficiencies in the pathway for clinician researcher careers

In the third main theme, clinician researchers discussed the lack of clear career structure and pathway for progression for research in health services: “I wouldn't have said at the start that I felt there was a structure that I could aim for” (P14). This had negative implications for career outcomes, as P28 outlined: “I actually feel like [reducing clinical load to do research] almost hindered my career progression, because there is not yet that clear research pathway.”

One of the ways in which the interviewees perceived the career pathway as deficient was a lack of shared understanding of what is expected of different levels of the existing pay structures in health. This applied to both determining the level of pay for research-focused positions, but also research expectations for primarily clinical roles. These expectations were sometimes linked to the person who occupied the role, rather than being a consistent expectation of clinicians at that level.

Participants also indicated that there was an absence of shared understanding of career structure for clinician researchers in health services to help guide their progress and career planning. They noted that there is a clear structure for clinicians (e.g. graduate, junior, senior, advanced, leadership) and for researchers in university settings (e.g. research fellow, senior research fellow, associate professor, professor). Participants noted that neither structure maps directly to clinician researchers, who might have less clinical and research experience at each level as a consequence of pursuing both skill sets.

Some participants noted that current awards in health services typically do not allow progression through promotion, and instead rely on positions becoming vacant. This was highlighted by P35, who expressed frustration that “there's no way for me to go to a [higher-level position] in the future, without those positions becoming vacant.” This was expressed as a limitation in the career structure for research, especially contrasted with university systems, which often allow staff to apply for a change in level based on experience. This barrier to career progression is compounded by the fact that many participants were already in senior or advanced roles, and by a ceiling effect in AH in general.

A common issue raised by participants was a gap in the career pathway for clinician researchers directly after completing a PhD, when there was limited direction or opportunities. P13 captured this by saying that after PhD conferral “there is a real ‘Well, what next? What next?’” There was a strong feeling that many get “lost” from a clinician researcher career at this point due to this gap. Participants pointed to a lack of postdoctoral type roles within health services, and a lack of suitability of postdoctoral roles available in universities for people who want to maintain a clinical or health service role. This was despite the fact that more AH clinicians are undertaking PhDs.

Gaps were also identified at other levels. Some participants felt that while there were currently reasonable opportunities to get involved in research for entry level clinicians, there were very few actual positions with a substantial research component at this level in health services. P33 referred to entry-level roles, saying “even from the beginning, the pathway is not there.” Participants also occasionally noted a lack of higher level roles in the health service, for example between senior research fellow and Professorial level.

#### Theme 4: Clinician researchers are not always valued or incentivized by health services

The fourth theme identified was that clinician researchers felt that their career path was not always incentivized or valued in health services in terms of career opportunities, support and progression. While some participants noted that they were incentivized and valued in specific teams and by specific managers, they usually did not feel that this was the norm across health services. P1 outlined this by stating, “I think it is [valued]. But again, I think it that depends on who's in [the position] at the moment, and also who the manager is at the moment…If someone else came into my manager's job and said, ‘I don't really care about research’, I wouldn't really have the opportunity.”

As noted in Theme 3, participants expressed the opinion that the only way to progress in a career in health services is to apply for higher-level roles. Clinician researchers felt that the value of their research experience in the recruitment process varied between health services, and often “depends on the manager, what they value the most” (P40). In some settings, research experience was not considered relevant to career progression in health. In other settings, research experience was considered valuable for progression to higher levels, but was nevertheless rarely considered necessary. As P12 said, “People value [PhDs] in academic worlds, but not in the hospital system. It doesn't help you get a job.”

However, there were other less traditional benefits to having research experience for job-seeking. Clinician researchers often felt that their research experience had opened up alternate career pathways, often in terms of lateral moves rather than progression. P32 said, “I definitely feel that the research skills have been the big selling point for me to leverage off to be able to move laterally.” A key driver of these opportunities was that research often enabled networking with high-level staff in health services. Participants also believed that the transferable skills (e.g. project management and critical thinking) they had gained would be valuable in a range of positions, but also felt that this was not always recognized in the hiring process.

Another factor contributing to participants’ perception of not being valued was the fact that, in general, clinical research positions were considered less desirable than clinical roles. Pay was considered to be sometimes inferior, especially for university-administered roles. While roles administered through the health service were generally in line with clinical salaries, many of the positions or options for combining research with clinical practice were less stable (i.e. grant funding, contract positions). Some participants felt that the unique skill mix of clinician researchers was not reflected in pay level, as their amount of experience was compared unfavourably to either full-time clinicians or researchers. As P10 outlined, “You almost shoot yourself in the foot in both camps, because you're not doing either one full time.”

In particular, participants noted the significantly reduced income from a clinician salary to a PhD stipend as a disincentive for pursuing research. Research degrees were not seen as a sound financial investment, as there was no corresponding pay increase in line with the skills gained. Many participants mentioned that although they did not pursue research for financial reward, they often felt disappointed in the lack of financial incentives. P12 said, “We don't do things for the money, but it actually is still nice to see career progression and just move and progress, that whole idea of just building and moving and not being stuck.”

Some participants noted that there were considerable barriers to succeeding in their clinician researcher roles in health services, linked to a perceived lack of value by the health service. Some participants felt that research was greatly valued in their department, some felt it was not valued at all, and some felt it was given a superficial value but that true support was lacking. This had a significant impact on career satisfaction and incentive to continue a research career in health services. Barriers as straightforward as not being able to travel internationally (without substantial paperwork and processes) to present research, or not being allowed to use grant money due to lack of backfill were frustrations that contributed to a general feeling that their role was not valued or supported. A few clinician researchers even described feeling “guilty” for taking time to do research, and that colleagues saw this as failing to help with clinical loads.

#### Theme 5: The current career challenges impair the viability of clinician researcher careers

Despite a desire for clinician researcher careers, the lack of extrinsic drivers outlined in the themes above (i.e. lack of jobs, unclear career structure and lack of career incentives) meant that many participants felt that maintaining a clinician researcher career was difficult. As P3 stated, “I just really value doing research at a clinical site. But how long I can do that in the current environment is a constant unknown.”

The outcome of this was that clinician researchers often had to divert towards either academia or clinical practice.

While all participants were currently engaged in research in a health service, they often spoke about the difficulty of maintaining this dual role. Participants described seeing other clinician researchers leave their health service or stop engaging in research, and felt this was a possible outcome for them. As P10 stated, “You're either a clinician or you leave to go work at a university.” They spoke about colleagues who had left the health service for a university setting, as it was the only viable way for them to engage significantly in research. Other colleagues, even those who had completed a PhD, went back to entirely clinical roles, sometimes attempting to pursue research in their own time, which was associated with reduced morale. In the respondent validation, P5 noted that she had already left health services for a university role since her interview, primarily because of the lack of stability of her position.

In a few cases, participants spoke of more positive outcomes, but acknowledged this was rare. For example, P55 mentioned, “In the hospital network I work in, most of them do come back [after PhD], and most of them are clinicians as well. Which is why I think I'm really lucky, because for me, that is my ideal. But majority of the time, I don't know that that happens.”

As a result of difficulty maintaining this dual role, participants felt that the unique combined skill set of clinician researchers was being lost. As P15 outlined, “If you're faced with having to choose between a research career or clinical career, then one or the other is going to lose out; you're not using the full array of your skill set.” This was seen as a negative outcome for both research and patient care. It was felt that moving into a full-time research position was a waste of clinical skill that was often quite advanced, while returning to a full-time clinical role was seen as not utilizing research skills that were often quite demanding to acquire in the first place.

#### Theme 6: The clinician researcher career path has been improving, and there is hope it will continue to improve

The final theme was a singular issue, reoccurring through many interviews, and related to hope for the future. While many participants felt that the current situation for clinician researcher careers was poor, they also felt this was improving. Many participants stated that they had personally seen the situation improve, especially in the past 5 years. They felt that during this time, more jobs had become available, research had begun to become an accepted part of clinical roles, and research skills were more valued in the career structure. Participants were cautiously hopeful that this positive trend would continue into the future, but acknowledged that the pace of change was likely to be slow, especially when it came to formation of new positions.

On the other hand, some participants also expressed scepticism and frustration at the slow pace of change, like P51, who said, “I can't help but have that pessimistic voice on my shoulders saying…I've seen this go in a cycle, both in Australia and New Zealand, and not a lot of change occurring.” During respondent validation, P17 also noted that sense of hope may be affected by location, saying, “I have not seen evidence of [hope] in rural and remote health services.” A sense that things will improve was seen as vital for current clinician researchers’ morale, as P7 noted: “I think you need that little sense of hope of a good outcome. Otherwise, some of us would just drop the whole research thing and look for a different career altogether.”

## Discussion

Overall, the participants of this study felt that AH clinician researcher careers are underdeveloped in Australia. While clinician researchers wanted to stay employed in hybrid positions in health services, this is jeopardized by a lack of jobs, an unstable career pathway and a lack of valuing or incentivizing of their career choice. This means that many are unsure whether they will be able to continue in this career, risking loss of the known value that these positions bring to health services.

This study’s finding that participants described a lack of clinician researcher roles, and desired more, is unsurprising. Clinical roles which include time for research and dedicated clinician researcher roles emerged as an important driver of research capacity-building in AH in two reviews [25, 26]. However, beyond recommending more roles, there has been little guidance from the literature on how many positions are needed, what types of positions, and how to generate them. From this research, it emerged that there was no single type of desired “dream” position (Subtheme 1.3). Thus, a range of position types may be beneficial for the career pathway, including those that are majority clinical or majority research, and both health service only and health service–university conjoint roles.

The participants’ view that there is a particular paucity of postdoctoral opportunities, as outlined in Subtheme 3.4, has also been supported by the literature. This has been illustratively referred to as the “postdoctoral clinical–academic void” [33, p. 54] and a “cliff edge on the pathway” [23, p. 6] by other authors. There has been some empirical research supporting this, although the issue has not been described in depth [34]. A survey of an Australian health service found that 25% of their (multidisciplinary) research-inactive staff held a PhD, demonstrating underutilization of this qualification and associated skills [12]. Another survey study found that the majority of American dieticians with PhDs worked in universities, while only 6% worked in clinical settings [35]. This gap has also been recognized for postdoctoral nursing, midwifery and AH clinicians in the United Kingdom, and strategies are currently being implemented to engage and capitalize on this workforce [33]. Thus, this appears to be an international challenge, and one in which successful approaches in other countries could be adapted for the Australian context.

The findings of this study also support the notion that clinician researcher careers are not perceived as extrinsically rewarding, detailed in Theme 4. Other research has touched upon this issue, but not to the level of focus of the current study. A survey of American AH professionals who had an interest in research found that only 3% listed financial compensation as a motivator for doing research, and 20% felt that research did not sufficiently reimburse them for their time [36]. Multiple qualitative studies have found that expectation of low pay is a disincentive for pursuing research careers, but this was often with respect to PhD stipends rather than general salaries [23, 34, 37, 38]. In some studies, a perceived lack of stability of clinician researcher positions was also briefly noted but not elucidated [23, 39, 40]. Another form of extrinsic reinforcement found to be lacking in the current research was a feeling of being valued by the heath service. One study on psychotherapists found that a lack of extrinsic reinforcement for combining research and practice was a challenge to this career [38]. This also echoes the idea highlighted in Subtheme 2.2 that clinician researchers are forced to make their own career opportunities.

In the current study, there were mixed opinions amongst respondents about the role of research in career advancement in health services (Subtheme 4.1). Most felt that research experience was valuable in some ways in some contexts by some managers, but very few felt there was consistency. Many felt that the “soft skills” gained in pursuing research were highly useful in many types of roles, but again were not consistently recognized. Similar conflicting findings have been reported in other studies. Survey studies have consistently identified “career advancement” as one of the top three motivators for AH clinicians to engage in research [41–45]. Contrasting this, a survey study of registered dieticians found that 40.4% disagreed or strongly disagreed that research is associated with career advancement at their place of employment [46], a concern which qualitative studies have also touched upon [34]. One explanation for this is that the value of research, and hence its role in career advancement, may differ among different professions, geographical locations or types of workplace. Another possibility is that the notion of “career advancement” has different interpretations—some may interpret it as developing their skill set, while others interpret it as pursuit of higher-level positions. Despite contradictory results in the literature, it is clear that for clinician researcher careers to be viable within health services, research must be perceived to have value for career development.

The finding that the deficiencies in the clinician researcher career pathway create pressure to abandon this dual career (Theme 5) is reflected in the literature, though mainly in reference to the postdoctoral period. The study of nursing, midwifery and AH clinical academics in the United Kingdom referred to in the introduction [23] found that “[they] face a decision to return to their pre-PhD clinical role (and hence not have their academic skills recognized and utilized) or follow a traditional academic research pathway and leave their clinical post behind (thus negating the whole reason for pursuing a clinical academic career)” [23, p. 6]. The study also noted that there are limited opportunities to develop a parallel clinician researcher career in the United Kingdom, and that clinicians are usually expected to achieve substantial clinical experience before weaving research into their career. Deficiencies of the clinician researcher career pathway from early career onwards are also present in Australia [6], as outlined in Theme 3 of this study. This has been previously identified as an issue in survey studies, where organizational success in “ensuring staff career pathways are available in research” has been consistently rated poorly by AH clinicians [19, 41, 42, 45, 47, 48]. A qualitative study of senior AH managers in the state of Queensland identified a lack of career pathways in research as a key challenge for research capacity-building [49]. Author commentary in the literature has consistently stated that career pathways are needed in AH. However, there has been little characterization beyond identifying this as a need, a gap which this research has helped to address.

While it is evident that other research supports the findings of this study, no other study has explored all of the elements of the clinician research career pathway as a focus. Existing research has touched upon elements of our findings in a non-systematic way–for example a single question in a survey study, or a single theme or quote in a qualitative study. This study is unique in focusing on describing career needs and challenges from the perspective of clinician researchers. As such, the study helps to bring together the threads present in the literature into a coherent picture to better understand, and therefore support, clinician researcher careers. As much of the literature in AH to date has focused on initial engagement of clinicians in research, rather than what comes after, these findings are important for growing clinician researcher capability in Australia and internationally.

Social cognitive career theory is a possible lens through which to examine and interpret the overall findings of this research [50]. This theory posits that career interests are formed from an individual’s interests (what career they think will be interesting), their self-efficacy expectations (how good they think they will be at the career) and the outcome expectations (what they think they can gain from that career) [50]. All of these elements combine with environmental barriers and facilitators to form an individual’s actual career choices and whether they are able to pursue the career they are interested in. This study demonstrated that despite a high level of individual interest in clinician researcher careers [12, 51], there is a low level of outcome expectations for engaging in this career path, potentially affecting clinicians’ interest in this career in the first place. Furthermore, the current study found that there were limited opportunities to engage in hybrid careers and a lack of external rewards, meaning that even if interest is high, actual pursuit of this career is compromised. Clinician researchers persist in pursuing these careers mostly because of personal interest and perceived benefits for their patients and health service delivery. In essence, while intrinsic drivers for pursuing clinician research careers are substantial, extrinsic drivers in Australia are currently poorly developed.

Despite much discussion of the many limitations of the current career opportunities and pathways available for clinician researcher positions, the results of this study also revealed that respondents felt that things were improving (Theme 6). This was particularly apparent amongst Queensland participants, possibly as a result of substantial investment in the past 10 years [13, 14]. This research also showed that many had hope it would continue to improve. However, it should be noted that many interviews were conducted before the budgetary impacts of COVID-19 had significantly affected Australian health services. It is yet unclear what the effects of this pandemic might be on the clinician researcher workforce and opportunities [52]. Nevertheless, this research has helped to delineate gaps and potential improvements in the current pathway, from the perspective of clinician researchers.

Policy-makers in both health services and academia should consider the economic and social implications of underutilizing the skill set of these professionals after training them for upwards of 8 years. It is clear that more AH positions that not only allow, but value, the combination of clinical and research work are needed in Australian health services. This is particularly true for the postdoctoral period. In addition to availability of positions, the extrinsic reward system in terms of financial reward and career progression also needs further attention from health services to ensure these careers are appealing. Academic policy-makers need to partner with health services to create more stable funding streams for AH professionals who wish to maintain clinical work while undertaking PhDs. It should also be noted that clinician researcher careers are relatively new to the AH workforce, and other research has described the challenges in navigating the formation of this professional identity [53]. Thus individuals may benefit from structured peer networks and mentorship programmes that help them navigate some of the unique challenges associated with these careers.

### Limitations

As with any qualitative research, the findings are representative of the individuals interviewed, and transferability of findings cannot be ascertained. The study did not use any data triangulation to verify participants’ statements (collect information about salaries, verify whether more AH clinicians are doing PhDs, etc.). While purposive sampling was used to capture a range of professions and states, the sample had overrepresentation of speech pathologists, Queensland-based participants and public metropolitan health services. Without a broad-scale understanding of the demographics of clinician researchers, it is unclear whether this sample is truly representative of the population—for example, whether clinician researchers with these characteristics are simply more common. This is another focus for future quantitative research. Although the same recruitment process was used for each state, it is likely that the recruitment was influenced by the location of the researchers in Queensland, as participants are generally more likely to participate when they recognize the researchers involved. Similarly, the fact that both researchers were speech pathologists likely contributed to the strong participation from this profession. Low sampling of participants from the state of Victoria was also potentially impacted by the substantial COVID-19 lockdown that was in place in that state at the time of recruitment [54].

## Conclusions

This research demonstrated that clinician researchers in Australia feel there is a lack of extrinsic drivers for pursuing this dual career. The combined effect of a lack of jobs, an unstable career pathway and a lack of valuing or incentivizing their career choice means that this career pathway is at risk. Many of the individuals interviewed in this study felt they might need to either divert to research in a university setting, or go back into full-time clinical practice. While the value of having university academics with clinical backgrounds and clinicians who are research-trained cannot be overstated, there was a sense that this underutilizes the unique combined skill set of these individuals. This work highlights potential targets for improvements to the career pathway for Australian and international health services and other relevant stakeholders.

## Supplementary Information

**Additional file 1.** Interview questions.

## Data Availability

The datasets generated and/or analysed during the current study are not publicly available due to the identifiability of the interviews, even with considerable redaction. Portions of interviews may be made available from the corresponding author on reasonable request.

## Abbreviations

AH: Allied health
