# Supplementary material for: “There hasn’t been a career structure to step into”: a qualitative study on perceptions of allied health clinician researcher careers
Source: Health Res Policy Syst. 2022 Jan 9;20:6. doi: 10.1186/s12961-021-00801-2 (PMC8743061; doi:10.1186/s12961-021-00801-2)
Supplement: Supplementary file 1 — Additional file 1. Interview questions. [file 12961_2021_801_MOESM1_ESM.docx]

**Appendix A: Interview Questions**

1. Prior to your current position, what has been your career pathway to date, and how has clinical research been able to be incorporated into those previous roles?

2. Please describe your current role, and then explain how the position is structured to enable you to conduct clinical research while in the role?

3. In a perfect world, what would be 2 or 3 ways you would improve your current position to better support/facilitate your clinical research activities?

4. Would you ever consider seeking other job opportunities outside health in order to pursue your research activities? And explain why?

5. If you could be in your “dream position”- what would that look like for you?

6. Do you feel there is a career structure/career pathway in health for people who want to do both clinical practice and clinical research in their role? Please explain your answer

7. How do you see your research activity/performance contributing to future career opportunities in health for you?

8. What are your thoughts about the statement “Senior leadership positions in health should be held by people who have both advanced clinical and research experience”

9. Do you have any other suggestions to help improve jobs, career opportunities, and/or the career pathway to support people who want to be active clinical researchers within health?
